
JOURNAL INFORMATION
==============================
NLM Title Abbreviation: ChemistryOpen
Journal ID: ChemistryOpen
NLM Journal ID: 101594811
Journal ID: open

ChemistryOpen

EISSN: 2191-1363
Publisher: Wiley

ARTICLE INFORMATION
==============================
PMCID: PMC3775516
PMID: 24551551
DOI: 10.1002/open.201390016
Article version: 1
Subjects: Graphical Abstract

Graphical Abstract: ChemistryOpen 4/2013

Print publication date: 2013 Aug
Electronic publication date: 2013 Aug 26
Volume: 2
Issue: 4
First page: 127
Last page: 130
Copyright: Copyright © 2013 WILEY-VCH Verlag GmbH & Co. KGaA, Weinheim
Copyright year: 2013
License: Re-use of this article is permitted in accordance with the Creative Commons Deed, Attribution 2.5, which does not permit commercial exploitation.
License URL: https://creativecommons.org/licenses/by/2.5/

==============================
### ChemistryOPEN

Including Thesis Treasury

04/2013

ChemistryOPEN... is a multidisciplinary, gold-road, open-access, international forum for the publication of Full Papers and Communications from all areas of chemistry and related fields. *ChemistryOpen* also publishes the Thesis Treasury containing summaries of Ph.D. theses and links to the full version via our homepage. Based in Europe, *ChemistryOpen* attracts authors and readers from around the world, as open-access publishing becomes more important in all areas of chemistry. *ChemistryOpen* is co-owned by ChemPubSoc Europe and published by Wiley-VCH. Authors can submit their primary research articles and thesis summaries via our homepage by clicking "submit an article". All contributions considered suitable for publication are subject to peer review, and if accepted, electronically processed and published online ensuring high quality and short publication times.

OPEN

Discover this journal online at:

WILEY ONLINE LIBRARY

wileyonlinelibrary.com

Most of the articles in this issue have already appeared online on wileyonlinelibrary.com.
See http://www.chemistryopen.org under EarlyView®

### COVER PICTURE

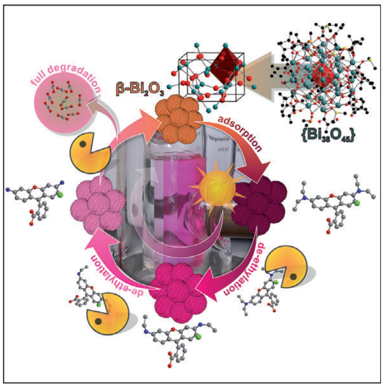

**The cover picture shows** the degradation of the model dye rhodamine B (RhB) in water using β-$Bi_2O_3$ nanoparticles as photocatalyst, which was prepared from preorganized bismuth oxido clusters. In the background, a homemade photoreactor for in situ studies is shown, including the decolorizing process of the aqueous RhB solution as observed during visible light irradiation. The catalytic cycle shows crucial steps in RhB degradation at the surface of β-$Bi_2O_3$ nanoparticles (balls), which can be recycled. After adsorption of RhB at the surface of β-$Bi_2O_3$ nanoparticles, the color of the nanoparticles changes from orange to violet. The degradation process (represented by Pac-Man) is induced by irradiation with visible light, resulting in a complete decolorization of the solution as well as the surface of the β-$Bi_2O_3$ nanoparticles, indicating a fast de-ethylation process. The latter process is faster than desorption of partially degraded RhB species from the β-$Bi_2O_3$ surface, but is demonstrated to occur upon irradiation of RhB-loaded β-$Bi_2O_3$ in the solid state. For more details, see the Full Paper by Michael Mehring et al., on p. 146 ff.

### NEWS

Spotlights on our sister journals *132 – 135*

WILEY ONLINE LIBRARY © 2013 Wiley-VCH Verlag GmbH & Co. KGaA, Weinheim

ChemistryOPEN Masthead

ChemPubSoc Europe www.chemistryopen.org

Gesellschaft Österreichischer Chemiker
Austria
Belgium
Czech Republic
Société Chimique de France
France
GDCh
Germany
Greece
Hungary
Italy
KNCV
Netherlands
Poland
Sociedade Portuguesa de Química
Portugal
Slovakia
Real Sociedad Española de Química
Spain
Svenska Kemistsamfundet
Sweden
SCS Swiss Chemical Society
Switzerland

### Editorial Advisory Board

### *Chairmen*
**Ramón Martínez-Máñez (Spain)**
**Thomas Wirth (UK)**

### *Honorary Chairman*
**Jean-Marie Lehn (France)**

### *Members*
Fernando Albericio (Spain)
Valentin Ananikov (Russia)
Didier Astruc (France)
Jochen Autschbach (USA)
Martin Banwell (Australia)
Vincenzo Barone (Italy)
F. Matthias Bickelhaupt (The Netherlands)
Kathrin Breuker (Austria)
Young Keun Chung (South Korea)
Richard G. Compton (UK)
Ulf Diederichsen (Germany)
Lothar Dunsch (Germany)
Antony Fairbanks (New Zealand)
Ernest Giralt (Spain)
Stefan Grimme (Germany)
Dirk Guldi (Germany)
Akira Harada (Japan)
Narayan Hosmane (USA)
Ken Houk (USA)
Osamu Ito (Japan)
Christoph Janiak (Germany)
Kazunori Kataoka (Japan)
Yoshiaki Kiso (Japan)
Mats Larhed (Sweden)
Lechoslav Latos-Grażyński (Poland)
Thisbe Lindhorst (Germany)
Luis M. Liz-Marzán (Spain)
Carlos Lodeiro (Portugal)
Todd L. Lowary (Canada)
Jean Martinez (France)
Pedro Matias (Portugal)
Stefan Matile (Switzerland)
Pedro Molina (Spain)
K. C. Nicolaou (USA)
Berit Olofsson (Sweden)
Oliver Reiser (Germany)
Kari Rissanen (Finland)
Knut Rurack (Germany)
James Rusling (USA)
Albert Schenning (The Netherlands)
Robert Schlögl (Germany)
Patrick Schmuki (Germany)
Ulrich S. Schubert (Germany)
Min Shi (China)
Norio Shibata (Japan)
Rint Sijbesma (The Netherlands)
Choong Eui Song (South Korea)
Petr Štěpnička (Czech Republic)
Brian Stoltz (USA)
Steve Suib (USA)
Reshef Tenne (Israel)
Matthew Todd (Australia)
Claudio Toniolo (Italy)
Jaume Veciana (Spain)
Hans-Achim Wagenknecht (Germany)
Ralph Weissleder (USA)
Gunther Wittstock (Germany)
Vivian W. W. Yam (China)
Chun-Hua Yan (China)
Jincai Zhao (China)

**Open Access and Copyright**

All research articles published by *ChemistryOpen* are fully open access: immediately freely available to read, download and share. All *ChemistryOpen* articles are published under the terms of the Creative Commons Attribution Non-Commercial License which permits use, distribution and reproduction in any medium, provided the original work is properly cited and is not used for commercial purposes.

Copyright on any research article published by *ChemistryOpen* is retained by the author(s). Authors grant Wiley a license to publish the article and identify itself as the original publisher. Authors also grant any third party the right to use the article freely as long as its integrity is maintained and its original authors, citation details and publisher are identified.

**Use by commercial “for-profit” organisations**

Use of Wiley Open Access articles for commercial, promotional, or marketing purposes requires further explicit permission from Wiley and will be subject to a fee. Copyright 2012 Wiley-VCH Verlag GmbH & Co. KGaA, Weinheim. All rights reserved. Requests to reproduce material from this journal content should be addressed to rights@wiley-vch.de, and reprints of *ChemistryOpen* articles can be purchased from chem-reprints@wiley-vch.de.

**Disclaimer**

The Publisher and Editors cannot be held responsible for errors or any consequences arising from the use of information contained in this journal; the views and opinions expressed do not necessarily reflect those of the Publisher and Editors, neither does the publication of advertisements constitute any endorsement by the Publisher and Editors of the products advertised.

Wiley Open Access articles posted to repositories or websites are without warranty from Wiley of any kind, either express or implied, including, but not limited to, warranties of merchantability, fitness for a particular purpose, or non-infringement. To the fullest extent permitted by law Wiley disclaims all liability for any loss or damage arising out of, or in connection with, the use of or inability to use the content.

**Editors:**
Karen Hindson, Haymo Ross

**Deputy Editor:**
Natalia Ortúzar

**Associate Editor:**
Saskia Neubacher

**Secretary:**
Vanessa Müller

**Production:**
Stefanie Klein, Margitta Schmitt

**Telephone:**
(+ 49) 6201-606-305

**Fax:**
(+ 49) 6201-606-203

**E-mail:**
chemistryopen@wiley-vch.de

**Courier Services:**
Boschstrasse 12
69469 Weinheim (Germany)

**Regular Mail:**
Postfach 10 11 61
69451 Weinheim (Germany)

**Homepage:**
http://www.chemistryopen.org

**Manuscript Submission & Personal Homepage**
manuscriptXpress
**http://www.manuscriptxpress.com**

**Customer Services:**
www.wileycustomerhelp.com

**Marketing:**
Monika Silz (ext. 458, fax: ext. 100,
e-mail: msilz@wiley-vch.de)

**Advertising:**
Marion Schulz (ext. 565, fax: ext. 550,
e-mail: mschulz@wiley-vch.de, jspiess@wiley-vch.de)

**Copyright Permissions:**
Bettina Loycke (ext. 280, fax: ext. 447,
e-mail: rights@wiley-vch.de)

**Reprints, e-prints, posters:**
Carmen Leitner (fax: ext. 226)
e-mail: chem-reprints@wiley-vch.de

**Publishers:**
WILEY-VCH Verlag GmbH & Co. KGaA,
Postfach 10 11 61
69451 Weinheim (Germany)
http://www.wiley-vch.de/

**Publication:**
6 issues in 2013

© 2013 WILEY-VCH Verlag GmbH & Co. KGaA,
Weinheim.

### COVER PROFILE

*"Water, the most important resource worldwide, is becoming more and more contaminated by chemical products, but established water purification processes are not exhaustively accessible. There is a strong need to develop cheap, efficient and decentralized water purification processes that make use of alternative energy sources."* More about the story behind the cover research can be found on p. 146.

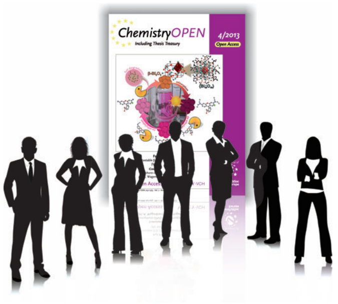

*M. Schlesinger, M. Weber, S. Schulze, M. Hietschold, M. Mehring**

***126***

**Metastable β-$Bi_2O_3$ Nanoparticles with Potential for Photocatalytic Water Purification Using Visible Light Irradiation**

### COMMUNICATIONS

**A two-way street:** The photochemically active chromophore–nucleic acid conjugates presented herein allow not only imaging by fluorescence microscopy but additionally are able to photoinduce 3,3′-diaminobenzidine (DAB) polymerization for staining to enable additional readout with electron microscopy.

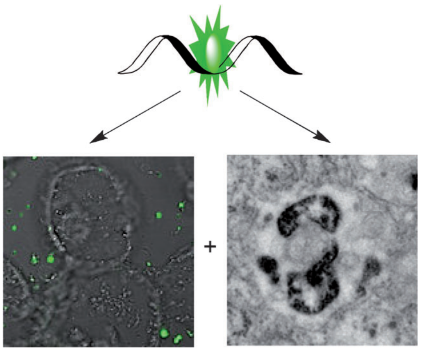

*C. Holzhauser, S. Kracher, M. M. Rubner, W. Schmucker, H.-A. Wagenknecht,* R. Witzgall**

***136 – 140***

**Photochemically Active Fluorophore–DNA/RNA Conjugates for Cellular Imaging of Nucleic Acids by Readout in Electron Microscopy**

**Off-line amplification:** A low-cost inkjet-printed gold nanoparticle immunoarray was integrated into a microfluidic device for multiple protein determinations. The use of heavily labeled magnetic beads allows optimization either for ultrasensitive detection of biomarker proteins (low fg $mL^{-1}$ levels) or for rapid 8 min assays within clinically relevant detection ranges.

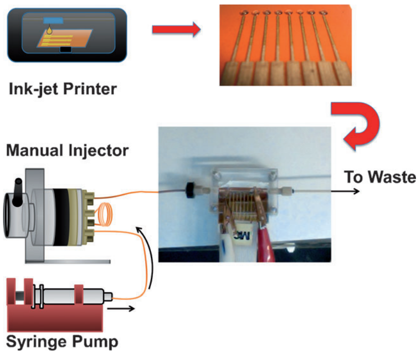

*C. E. Krause, B. A. Otieno, A. Latus, R. C. Faria, V. Patel, J. S. Gutkind, J. F. Rusling**

***141 – 145***

**Rapid Microfluidic Immunoassays of Cancer Biomarker Proteins Using Disposable Inkjet-Printed Gold Nanoparticle Arrays**

© 2013 Wiley-VCH Verlag GmbH & Co. KGaA, Weinheim

### FULL PAPERS

M. Schlesinger, M. Weber, S. Schulze, M. Hietschold, M. Mehring*

*146 – 155*

**Metastable β-$Bi_2O_3$ Nanoparticles with Potential for Photocatalytic Water Purification Using Visible Light Irradiation**

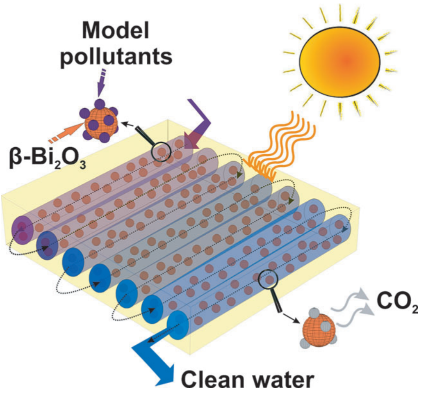

**Water purification with β-$Bi_2O_3$:** A straightforward synthetic procedure for the synthesis of β-$Bi_2O_3$ nanoparticles that offers the potential for gram-scale production is presented. The β-$Bi_2O_3$ nanoparticles show high photocatalytic activity and stability in the degradation of typical organic water pollutants under visible light irradiation. These nanoparticles might be promising materials for use in novel water purification systems.

J. Zhang, L. Zou, T. L. Lowary*

*156 – 163*

**Synthesis of the Tolerance-Inducing Oligosaccharide Lacto-*N*-Fucopentaose III Bearing an Activated Linker**

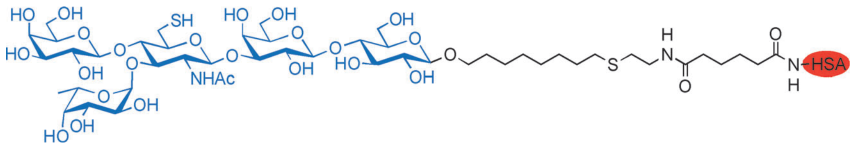

**(Synthetic) route to tolerance:** A concise synthetic route to a tolerance-inducing immunomodulatory pentasaccharide, lacto-*N*-fucopentaose III, and its corresponding human serum albumin conjugate is described. Key transformations include two highly regio- and stereoselective glycosylations, a Birch reduction, and a UV-promoted radical addition of a thiol to an alkene.

* Author to whom correspondence should be addressed.

Supporting information on the WWW (see article for access details).

This is an open-access article, published under the terms and conditions of the Creative Commons Attribution Non Commercial License.

A video clip is available as Supporting Information on the WWW (see article for access details).

VIP Contributions labeled with this symbol have been judged as "Very Important Papers" by the referees.

### SERVICE

**All the Tables of Contents may be found on the WWW under: http://www.chemistryopen.org**
